# Atlas and Epitome of Special Pathologic Histology

**Published:** 1900-12

**Authors:** 


ATLAS AND EPITOME OF SPECIAL PATHOLOGIC HISTOLOGY.—By Docent Dr. Hermann Durck, Assistant in the Pathologic Institute; Professor to the Municipal Hospital, Munich. Authorized translation from the German. Edited by Ludwig Hektoen, M. D., Professor of Pathology in Rush Medical College, Chicago. Circulatory organs, respiratory organs, gastro-intestinal tract. With sixty-two colored plates. 158 pages. Price, $3.00. Philadelphia: W. B. Saunders, publisher, 925 Walnut Street. 1900.

The profession is to be congratulated upon the receipt of this valuable translation. Two more volumes follow this, one completing special pathologic histology, the other dealing with general pathologic histology.

The illustrations in this work are from preparations, and the greatest care has been taken to make each picture absolutely exact.

